# Cross-lagged analysis of financial toxicity and psychological distress between breast cancer patients and spouses during chemotherapy: an actor–partner interdependence model

**DOI:** 10.3389/fpubh.2026.1838284

**Published:** 2026-06-30

**Authors:** Qing Wang, Lili Wang, Tingting Wang, Lili Li, Xiaoping Pang, Weijuan Yang

**Affiliations:** 1 Department of Breast Surgery, The First Affiliated Hospital with Nanjing Medical University, Nanjing, Jiangsu, China; 2 School of National Audit, Nanjing Audit University, Nanjing, Jiangsu, China

**Keywords:** actor–partner interdependence model, breast cancer, cross-lagged analysis, financial toxicity, longitudinal studies, nursing, psychological distress

## Abstract

**Aim:**

To explore the longitudinal interaction between financial toxicity and psychological distress in breast cancer patients and their spouses based on the actor-partner interdependence model.

**Methods:**

Convenience sampling method was used to select 258 breast cancer patients and their spouses as the research partners. The financial toxicity scale and distress thermometer were used to investigate the patients in the early stage (T1), the middle stage (T2) and the end of chemotherapy (T3).

**Results:**

The actor effects of financial toxicity on psychological distress were significant for both breast cancer patients and their spouses. Specifically, the level of financial toxicity in patients significantly predicted their own psychological distress at the next time point (T1 → T2: *β* = −0.19, T2 → T3: *β* = −0.18, both *p* < 0.001), and the level of financial toxicity in spouses significantly predicted their own psychological distress at the next time point (T1 → T2: *β* = −0.15, T2 → T3: *β* = −0.14, both *p* < 0.01). The actor effects of psychological distress on financial toxicity were also significant. That is, the level of psychological distress in patients significantly predicted their own financial toxicity at the next time point (T1 → T2: *β* = −0.18, T2 → T3: *β* = −0.17, both *p* < 0.01), and the level of psychological distress in spouses significantly predicted their own financial toxicity at the next time point (T1 → T2: *β* = −0.16, T2 → T3: *β* = −0.15, *p* < 0.01). The partner effects of psychological distress were significant. Specifically, patients’ psychological distress significantly and positively predicted their spouses’ psychological distress at the next time point (T1 → T2: *β* = 0.18, T2 → T3: *β* = 0.17, both *p* < 0.01), and spouses’ psychological distress significantly and positively predicted patients’ psychological distress at the next time point (T1 → T2: *β* = 0.20, *p* < 0.001; T2 → T3: *β* = 0.20, *p* < 0.01).

**Conclusion:**

The financial toxicity and psychological distress of breast cancer patients and their spouses have a two-way actor prediction effect.

## Introduction

Breast cancer is the leading malignant tumor that seriously threatens women’s health worldwide. As an important means of comprehensive treatment for breast cancer, chemotherapy not only improves the survival rate but also imposes a heavy physiological toxicity, psychological distress, and socioeconomic hardship (1, 2).

In recent years, with the application of precision medicine and new antineoplastic drugs, the cost of cancer treatment has risen sharply, and the concept of financial toxicity has become increasingly prominent (3, 4). Financial toxicity refers to the objective economic burden and subjective distress (5). Studies at home and abroad have confirmed that financial toxicity not only causes patients to delay or give up treatment and reduces the quality of life but also is an independent risk factor for anxiety and depression (6, 7).

In the care system of breast cancer patients, spouses usually play the core roles of caregivers and emotional supporters. During chemotherapy, patients experience multiple pressures, such as physical discomfort, image change, and uncertainty about the future, which easily lead to psychological distress like anxiety and depression (8–10). At the same time, spouses are also under great psychological and financial pressure while providing care, managing the family, and maintaining financial support (11). The actor–partner interdependence model (APIM) holds that there is a strong interpersonal interaction between partners in a close relationship, and individuals’ thoughts and behaviors are not only affected by themselves but also by each other (12). The mental health of patients and their spouses does not exist in isolation but continuously influences each other through the intimate relationship, forming a dynamic dyadic interaction system (13).

This study aims to focus on the dyadic core unit composed of patients and their spouses and deeply explore the internal interaction mechanism between them when coping with financial toxicity. Existing studies often simplify the spouse as a support source and fail to systematically reveal the interaction and temporal dynamics of the psychological distress of both parties under pressure.

Therefore, this study introduced the actor–partner interdependence model to dynamically track the change trajectory and predictive relationship between financial toxicity and psychological distress in breast cancer patients and their spouses during chemotherapy. By identifying the hysteresis effect of variables, the study is expected to clarify the critical intervention path and the time window, providing an empirical basis for the psychological support scheme for the dyadic unit to achieve integration and ultimately improving the common psychological adaptation and quality of life of patients and spouses.

We will conduct tracking from the early stage of chemotherapy until the end of chemotherapy and build a actor–partner model of intimacy. We propose the following hypotheses:

*H1*: The level of financial toxicity of patients (or their spouses) at one time point can positively predict their own level of psychological distress at the next time point (actor effect).

*H2*: The level of financial toxicity of the patient (or spouse) at a certain time point positively predicts the level of psychological distress of the spouse at the next time point (partner effect).

*H3*: There is also a cross-lag prediction effect between the psychological distress of patients and their spouses.

*H4*: There may be asymmetry in the mutual predictive relationship between financial toxicity and psychological distress between patients and their spouses.

## Participants and methods

### Participants

From July 2024 to June 2025, we selected 258 breast cancer patients and their spouses in the Department of Breast Surgery of The First Affiliated Hospital with Nanjing Medical University as actors through convenience sampling.

### Inclusion criteria

Patients who met the clinical diagnosis criteria of breast cancer and were (14)confirmed by postoperative pathology^[14]^ and received postoperative chemotherapy (docetaxel + cyclophosphamide, 4–6 cycles, with an interval of 21 days).

Both the patients and their spouses were 18 years old or older and had basic communication skills.

### Exclusion criteria

Patients undergoing palliative treatment, those with metastatic breast cancer, etc.

Patients with a history of mental illness.

### Withdrawal criteria

Participants who died during the follow-up period or did not complete three longitudinal measurements for other reasons.

According to the sample size formula of paired design $n={\left(\mu_{\alpha }+\mu_{\beta }\right)}^{2}\sigma_{d}^{2}/\delta^{2}$ and combined with the pilot study, the sample size should be at least 161 cases. Considering a 10% dropout rate in the longitudinal survey, the minimum sample size of this study was determined as *n* = 161 / (1–10%) = 179.

This study was conducted after obtaining the approval of the First Affiliated Hospital with Nanjing Medical University ethics committee (2024-SR-964).

All voluntarily participated in this study and signed the informed consent.

### Survey instrument

#### General information questionnaire

The general information questionnaire was compiled by the researchers, which included the basic information of the patients (age, education level, residence, residence style, economic income, tumor stage, whether to conserve breast) and the basic information of the spouses (age, education level, residence, economic income). To objectively assess household economic vulnerability, household income was categorized with reference to the local statutory minimum wage standard (2,020 yuan per month in 2021 for Nanjing, Jiangsu Province). All baseline data (T1) were collected prior to the first chemotherapy session.

#### Comprehensive scores for financial toxicity based on the patient-reported outcome measures (COST-PROM)

The scale was developed by DeSouza et al. and revised by Liu et al. (15). The scale consists of 11 items. Each item is assigned a score ranging from 0 to 4, and the total score ranges from 0 to 44. The lower the total score, the more serious the financial toxicity perceived by the respondents, and a score of <26 is defined as positive. In this study, the scale was used to investigate the financial toxicity of enrolled patients and their spouses.

#### Distress thermometer

The Distress Thermometer (DT) was used to assess a patient’s level of psychological distress over the past week (16). In this study, psychological distress was conceptualized as an umbrella term encompassing multidimensional suffering, extending beyond emotional reactions such as depression and anxiety to include imbalances across physical, social, and spiritual domains. The Chinese version of the Distress Thermometer (DT), adapted from the original NCCN instrument, was utilized in accordance with the NCCN Distress Management Guidelines (Version 1.2024). The accompanying Problem List captures five key dimensions: (1) Physical concerns (e.g., fatigue, pain, sleep); (2) Emotional concerns (e.g., anxiety, depression, fear); (3) Family/Social concerns (e.g., relationship with spouse, caregiving burden); (4) Practical concerns (e.g., financial problems, work/insurance); and (5) Spiritual/Religious concerns. In the Chinese cancer population, a cutoff score of ≥4 on the DT (which ranges from 0 [no distress] to 10 [extreme distress]) is recommended to indicate clinically significant distress, demonstrating a sensitivity of 0.88 and a specificity of 0.70. In this study, participants scoring ≥4 were classified as experiencing psychological distress (16).

#### Data collection methods

Before conducting the survey, all the researchers participating in the survey were trained. After obtaining the consent of hospitals and patients, a questionnaire survey was carried out, and the general information questionnaire was retrieved from the medical records of patients. The data of the financial toxicity scale and psychological distress scale were collected at the time points of pre-chemotherapy (before the first chemotherapy, T1), mid-chemotherapy (the second to third chemotherapy, T2), and the end of chemotherapy (the fourth to sixth chemotherapy, T3). The data at the T1–T3 time nodes were collected through face-to-face surveys in the ward. To ensure patient privacy, the survey was conducted anonymously in a confidential environment. Polygraph questions were added to the questionnaire to ensure its reliability and to guarantee that each questionnaire could reflect the true wishes of patients.

#### Statistical methods

SPSS 26.0 software was used for statistical analysis. The data were double-checked and entered. The measurement data that conformed to the normal distribution were expressed as (mean ± standard deviation), and the count data were expressed as the number of cases (percentage). Repeated measures analysis of variance was used to compare the scores of financial toxicity and psychological distress of patients and their spouses at three time points (T1–T3). The Pearson correlation test was used to analyze the correlation between variables. Mplus 8.3 software was used for longitudinal data analysis.

Firstly, to test the stability of the measurement tool at different time points, the configural equivalence model, factor loading equivalence model, and intercept equivalence model were constructed to test the measurement equivalence.

Secondly, a cross-lagged Actor–Partner interdependence model was constructed to test the interaction between financial toxicity and psychological distress of patients and their spouses at T1–T3. This model can simultaneously analyze the actor effect (i.e., the influence of the previous variable of the same individual on the subsequent variable) and the partner effect (i.e., the influence of the previous variable of one partner on the subsequent variable of the other partner).

The fit of the model evaluation standard is as follows: the comparative fit index (CFI) > 0.90, the Tucker– Lewis index (TLI) > 0.90, the root mean square error of approximation (RMSEA) < 0.08, and the standardized root mean square residual (SRMR) < 0.08.

## Results

### General information of respondents

The survey included 240 patient-spouse pairs, and their baseline demographic data are presented in Table 1.

**Table 1:** General information of respondents (*n* = 240).

Patient information		Number of cases (percentage, %)	Patient information		Number of cases (percentage, %)
Age (years)	< 45	67 (27.92)	Monthly income (yuan)	< 2,000	34 (14.17)
	45 ~	84 (35.00)		2,000~	71 (29.58)
	≥60	89 (37.08)		5,000~	75 (31.25)
Level of education	Junior high school and below	104 (43.33)		> 8,000	60 (25.00)
	High school	76 (31.67)	Tumor staging	I	102 (42.50)
	Junior College and above	60 (25.00)		II	90 (37.50)
Place of residence	Town	133 (55.42)		Iii and above	48 (20.00)
	Rural	107 (44.58)	Breast conserving or not	Yes	152 (63.33)
Mode of residence	Living alone	0 (0.00)		no	88 (36.67)
	Live with your family	240 (100.00)			

Spouse information		Number of cases (percentage, %)	Spouse information		Number of cases (percentage, %)
Age (years)	< 45	66 (27.50)	Place of residence	Town	133 (55.42)
	45 ~	85 (35.42)		Rural	107 (44.58)
	≥60	89 (37.08)	Monthly income (yuan)	< 2,000	14 (5.83)
Level of education	Junior high school and below	100 (41.67)		2000~	35 (14.58)
	High school	74 (30.83)		5,000~	114 (47.50)
	Junior college and above	66 (27.50)		> 8,000	77 (32.09)

#### Systematic error test for study design

The systematic error was measured by Harman’s single-factor test for common method deviation. This study adopted a longitudinal design, so the common method deviation of the data retrieved from 3 measurements was tested. The results showed that the variance interpretation rates of the first factor of the three measurements were 19.37, 23.42, and 25.33%, respectively, which were lower than the critical value of 40% (17). This indicates that there was no serious common method bias in this study.

#### Scores of financial toxicity and psychological distress of breast cancer patients and their spouses and their correlation analysis

The scores of financial toxicity of breast cancer patients and their spouses exhibited a significant downward trend over time (*p* < 0.001), while the scores of psychological distress showed a significant upward trend over time (*p* < 0.001), as presented in Table 2. The scores of financial toxicity and psychological distress were correlated during the T1–T3 stage, and this correlation was significant (*p* < 0.05), as shown in Table 3.

**Table 2:** Scores of financial toxicity and psychological distress of breast cancer patients and their spouses (*n* = 240, score, *−х* ± *ѕ*).

Items	Patient financial toxicity	Spouse financial toxicity	Patient psychological distress	Spouse psychological distress
T1	26.14 ± 3.40	27.07 ± 3.25	4.64 ± 1.19	4.04 ± 1.23
T2	25.47 ± 3.12	26.94 ± 3.39	4.99 ± 1.02	4.22 ± 1.14
T3	24.30 ± 3.39	26.11 ± 3.60	5.25 ± 1.27	4.85 ± 1.08
F	23.140	9.010	21.033	11.900
P	< 0.001	< 0.001	< 0.001	< 0.001

**Table 3:** Financial toxicity and psychological distress scores of breast cancer patients and their spouses and their correlation analysis (*r* value, *n* = 240).

Items	①	②	③	④	⑤	⑥	⑦	⑧	⑨	⑩	⑪	⑫
①	1											
②	0.599^**^	1										
③	0.510^**^	0.503^**^	1									
④	0.521^**^	0.435^**^	0.303^*^	1								
⑤	0.436^**^	0.467^**^	0.355^**^	0.522^**^	1							
⑥	0.403^**^	0.418^**^	0.412^**^	0.471^**^	0.407^**^	1						
⑦	−0.544^**^	−0.449^**^	−0.310^*^	−0.294^*^	−0.309^*^	−0.289^*^	1					
⑧	−0.477^**^	−0.457^**^	−0.350^**^	−0.342^**^	−0.357^**^	−0.335^**^	0.546^**^	1				
⑨	−0.400^**^	−0.414^**^	−0.428^**^	−0.376^**^	−0.336^**^	−0.441^**^	0.428^**^	0.505^**^	1			
⑩	−0.459^**^	−0.409^**^	−0.302^*^	−0.302^*^	−0.394^**^	−0.294^*^	0.472^*^	0.461^**^	0.285^*^	1		
⑪	−0.424^**^	−0.438^**^	−0.349^**^	−0.350^**^	−0.417^**^	−0.305^*^	0.404^**^	0.470^**^	0.306^*^	0.440^**^	1	
⑫	−0.363^**^	−0.394^*^	−0.391^**^	−0.415^**^	−0.421^**^	−0.394^**^	0.375^**^	0.385^**^	0.357^**^	0.381^**^	0.396^**^	1

① Patient financial toxicity in T1; ② Patient financial toxicity in T2; ③ Patient financial toxicity in T3; ④ Spouse financial toxicity in T1; ⑤ Spouse financial toxicity in T2; ⑥ Spouse financial toxicity in T3; ⑦ Patient psychological distress in T1; ⑧ Patient psychological distress in T2; ⑨ Patient psychological distress in T3; ⑩ Spouse psychological distress in T1; ⑪ Spouse psychological distress in T2; ⑫ Spouse psychological distress in T3.

**p < 0.01, *p < 0.05.

#### Test of the cross-time equivalence model of financial toxicity and psychological distress between patients and their spouses

The models of financial toxicity, configural equivalence, metric equivalence, and intercept equivalence of psychological distress of patients and their spouses were constructed respectively, and the degree of fit is shown in Table 4. The results of this study showed that the financial toxicity and psychological distress of patients and their spouses met the configural equivalence, metric equivalence, and intercept equivalence (∆CFI < 0.01) across time, which met the prerequisite for cross-lag analysis.

**Table 4:** Equivalence model fitting results of three measurements of financial toxicity and psychological distress for patients and their spouses.

Items		*χ* ^2^	*df*	*p*	CFI	∆CFI	TLI	RMSEA	SRMR
Patient financial toxicity	Configural equivalence	124.244	62	<0.001	0.990	—	0.985	0.038	0.023
	Metric equivalence	148.510	66	<0.001	0.990	<0.001	0.987	0.036	0.025
	Intercept equivalent	192.385	72	<0.001	0.984	0.004	0.979	0.042	0.027
Spouse financial toxicity	Configural equivalence	126.369	62	<0.001	0.990	—	0.984	0.039	0.031
	Metric equivalence	153.600	66	<0.001	0.990	<0.001	0.986	0.038	0.033
	Intercept equivalent	202.746	72	<0.001	0.985	0.003	0.983	0.039	0.035
Patient Psychological distress	Configural equivalence	216.593	102	<0.001	0.975	—	0.965	0.050	0.036
	Metric equivalence	244.808	110	<0.001	0.975	<0.001	0.968	0.047	0.037
	Intercept equivalent	314.422	118	<0.001	0.972	0.001	0.967	0.048	0.038
Spouse psychological distress	Configural equivalence	218.517	102	<0.001	0.973	—	0.959	0.055	0.055
	Metric equivalence	267.164	110	<0.001	0.971	0.001	0.964	0.054	0.055
	Intercept equivalent	330.220	118	<0.001	0.968	0.002	0.960	0.054	0.055

#### Longitudinal interaction between financial toxicity and psychological distress among breast cancer patients and their spouses

Establishment and fitting of financial toxicity and psychological distress models for breast cancer patients and their spouses.

In the model, the same observed variables at three measurement points were allowed to be correlated with the latent variable error at the same time point. Then, the model was restricted (model M2). That is, the autoregressive effect/actor effect and partner effect of patients and their spouses on the same variable were restricted to be equal. The actor effect and the partner effect were equal across different variables.

The results of model fitting are shown in Table 5. Compared with M1, M2 shows that ∆χ*^2^* (∆*df*) is significant, and ∆CFI ≤ 0.01, indicating that the difference between M2 and M1 is not significant. This indicates that there is no significant difference in the model fitting degree after applying the restriction, suggesting that the restriction is reasonable and the more parsimonious model M2 is accepted. Therefore, M2 was selected as the final model.

**Table 5:** The fitting results of the actor and partner interdependence model of financial toxicity and psychological distress for breast cancer patients and their spouses after limiting the model.

Model	*χ* ^2^	*df*	*p*-value	CFI	∆CFI	TLI	RMSEA	SRMR
M1: Saturation model of financial toxicity and psychological distress for breast cancer patients and spouses	2214.338	740	< 0.001	0.944	—	0.934	0.041	0.076
M2: Bounded model	2360.191	784	< 0.001	0.941	0.001	0.936	0.039	0.077

Path analysis of cross-lagged actor–partner interdependence model of financial toxicity and psychological distress of breast cancer patients and their spouses.

The financial toxicity and psychological distress of breast cancer patients and their spouses showed strong stability, and the autoregression coefficients (*β*) ranged from 0.38 to 0.52, with all *p* < 0.001. The higher the financial toxicity score, the lower the level of perceived financial toxicity.

The financial toxicity of breast cancer patients and their spouses has a significant actor effect on psychological distress. That is, the financial toxicity of patients and their spouses can positively predict their own psychological distress in the next stage.

The actor effect of psychological distress of patients and their spouses on financial toxicity is significant. That is, the psychological distress of patients and their spouses can positively predict their own financial toxicity in the next stage.

**Figure 1:**
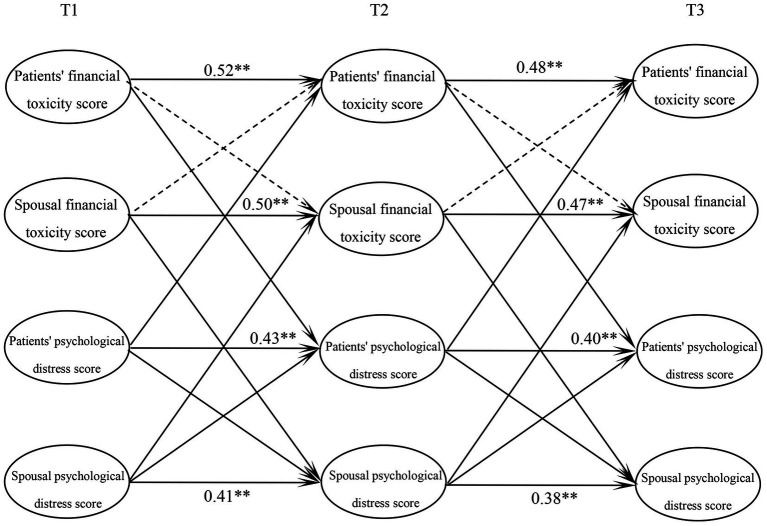
Cross-lagged actor–partner interdependence model of financial toxicity and psychological distress among breast cancer patients and their spouses. **denotes *P* < 0.001.

The partner effect of psychological distress is significant. That is, the psychological distress of patients and their spouses can positively predict the psychological distress of each other in the next stage, and the coefficients and paths are shown in Table 6. The standardized coefficients of the bounded model M2 are shown in Figure 1.

**Table 6:** Path analysis of cross-lagged actor–partner interdependence model for financial toxicity and psychological distress of breast cancer patients and their spouses.

Actor-partner effect	Path	*β* (T1 → T2)	*β* (T2 → T3)
Actor effect	Patients’ financial toxicity score → patients’ psychological distress score	−0.19**	−0.18**
	Spousal financial toxicity score → spousal psychological distress score	−0.15*	−0.14*
	Patients’ psychological distress score → patients’ financial toxicity score	−0.18*	−0.17*
	Spousal psychological distress score → spousal financial toxicity score	−0.16*	−0.15*
Partner effect	Patients’ psychological distress score → spousal psychological distress score	0.18*	0.17*
	Spousal psychological distress score → patients’ psychological distress score	0.20**	0.20*
	Patients’ financial toxicity score → spousal financial toxicity score	0.08	0.06
	Spousal financial toxicity score → patients’ financial toxicity score	0.08	0.06

*denotes *p* < 0.01, **p < 0.001.

## Discussion

The results of this study showed that the financial toxicity scores of breast cancer patients and their spouses during chemotherapy exhibited a significant downward trend over time. That is, the level of financial toxicity showed an upward trend, and the psychological distress scores showed a significant upward trend over time. The financial toxicity and psychological distress scores were correlated in the T1–T3 stage, and the correlation was significant.

The results indicated that the families of breast cancer patients were experiencing a double crisis (18) of economic burden and psychological distress during chemotherapy, which were intertwined and continuously worsened. The causes are multi-level.

At the partner level, with the progress of chemotherapy, the accumulated out-of-pocket medical expenses, the reduction in income caused by treatment or accompanying care, and the continuous increase in non-medical expenses (such as nutrition and transportation) directly exacerbate the family economic burden, that is, the level of financial toxicity increases (19).

At the psychological level, the reality of economic pressure can trigger and amplify the anxiety, helplessness, and uncertainty about the future of patients and their spouses. This escalated psychological distress may further worsen the economic situation by affecting treatment compliance, aggravating the perception of physical symptoms, or inducing avoidance/impulsive decision-making, thus forming an economy-psychological vicious circle (20).

In clinical practice, medical and nursing staff should establish a clinical pathway of regular screening—early warning–comprehensive intervention and systematically assess the economic risk and psychological distress level of patients’ families at the early stage of chemotherapy. Specific measures include introducing tumor financial navigation services, providing personalized cost management programs, including spouses in the psychological support system, and providing communication and coping skills training based on husband–wife units, so as to break the vicious circle of economic and psychological stress and improve the overall resilience of the family.

The cross-lagged analysis of this study revealed a significant bidirectional longitudinal predictive relationship between financial toxicity and psychological distress among breast cancer patients and their spouses. This confirms that economic pressure and mental health during the disease trajectory are not characterized by unidirectional causality, but rather constitute a dynamic, cyclical feedback system.

First, the pathogenic pathway from patient financial toxicity to psychological distress (Actor Effect). The findings indicate that patients’ financial toxicity at T1 significantly and negatively predicted their psychological distress at T2 (*β* = −0.19, *p* < 0.001). This suggests that with the accumulation of treatment costs, each unit increase in perceived financial toxicity significantly exacerbates subsequent symptoms of anxiety and depression. The underlying mechanism is as follows: Financial toxicity acts as a persistent deprivation stressor that directly erodes patients’ psychological sense of security and control. When faced with high out-of-pocket expenses or income interruption, patients experience intense fears regarding treatment sustainability. This objective financial predicament transforms into subjective existential anxiety, directly triggering clinically significant psychological distress.

Second, the pathogenic pathway from spouse financial toxicity to psychological distress (Actor Effect). The data show that spouses’ financial toxicity at T1 also significantly predicted their own psychological distress at T2 (*β* = −0.15, *p* < 0.01). This result implies that spouses are not merely providers of economic support but also primary bearers of economic pressure. In breast cancer families, spouses often bear the dual burden of maintaining household livelihoods and covering medical expenses. Their financial toxicity primarily stems from relative income deprivation (e.g., missed work, pay cuts) and absolute increases in expenditure. This depletion of financial resources directly weakens the spouse’s psychological resilience against the impact of the disease, resulting in chronic high pressure and a state of burnout. Consistent with the Stress Process Model (21), the cumulative burden of medical expenditures and income loss constitutes a financial predicament that significantly diminishes an individual’s sense of mastery and security (22, 23).

Third, the reverse exacerbation pathway from psychological distress to financial toxicity (Actor Effect). This study not only verified that financial toxicity leads to distress but also uncovered the reverse path: patients’ psychological distress at T1 significantly predicted increased financial toxicity at T2 (*β* = −0.18, *p* < 0.01). This indicates that severe psychological distress (anxiety, depression) can substantively worsen the economic situation. Patients in a state of high distress suffer from impaired cognitive function and tend to adopt avoidance coping strategies (e.g., delaying follow-up visits, refusing expensive but necessary tests) or irrational decision-making, leading to disease progression and increased long-term medical costs. Furthermore, psychological distress hampers executive function and risk judgment (24), leading individuals to make uneconomical medical choices that further inflate future costs (25, 26). The continuous depletion of psychological resources also reduces the willingness to seek external aid, thereby exacerbating the actual economic burden (27, 28). From the perspective of Family Systems Theory, the economic anxiety and psychological distress of patients and spouses reinforce each other through daily interactions, forming a closed negative feedback loop within the family^[29]^. Therefore, clinical interventions must break this cycle by establishing early, proactive, and integrated models combining psychosocial support with professional financial navigation.

Fourth, the dyadic spillover effect of psychological distress and cumulative family risk. Although financial toxicity did not show significant cross-actor predictive effects, psychological distress exhibited strong interpersonal contagion (Partner Effect). Patients’ psychological distress at T1 predicted spouses’ distress at T2 (*β* = 0.18, *p* < 0.01), and spouses’ distress at T1 similarly predicted patients’ distress at T2 (*β* = 0.20, *p* < 0.001). This implies that while both parties may carry their own separate economic burdens, they share a common emotional account. One party’s despair can rapidly deplete the other party’s psychological resources through emotional contagion and interactive conflict, leading to a decline in overall family resilience. Ultimately, the entire family system risks falling into a dual crisis of “financial bankruptcy” and “psychological collapse.” According to the theory of emotional contagion (29), persistent negative emotions in one party can be transmitted directly to the spouse, triggering empathic stress responses. As a highly interdependent emotional unit, psychological distress in either party may weaken the emotional support efficacy and communication quality within the family, easily triggering negative interaction patterns (e.g., demand-withdraw or mutual blame) (30, 31), thereby exacerbating the partner’s emotional burden and forming a mutually reinforcing stress cycle.

### Clinical implications

It is necessary to extend psychosocial intervention from the traditional individual level to the binary intervention model based on husband and wife. It is suggested that in clinical practice, the assessment of the psychological status of the spouse should be included in the routine screening system and incorporated into the systematic support plan together with the patient. By conducting participatory disease psychological education, couples communication skills training, and joint stress management strategies, the partner not only helps to block the cross-infection of negative emotions between the couple but also can promote the positive interaction between the two sides to build a collaborative response pattern to the disease. This can strengthen the function of the family as an emotional buffer system and overall promote the family’s psychological adaptation and adjustment in the process of responding to the disease.

### Limitations

This study has several limitations. First, the sample was recruited from a single center, and patients with metastatic breast cancer were excluded. This not only resulted in a homogenous sample in terms of cultural, economic, and medical support environments, limiting the generalizability of the findings across regions and different levels of healthcare institutions, but also likely led to an underestimation of financial toxicity, as the included patients generally had milder disease conditions compared to advanced cancer populations. Consequently, the generalizability of our conclusions to severely ill or end-stage patients is restricted.

Second, there are limitations regarding the demographic variables. While individual characteristics were collected, the study lacked consideration of dyadic relationship-specific features. Key variables such as marital duration, time since diagnosis, and the length of spousal caregiving were not included. As insightfully pointed out by the reviewer, particularly within the sociocultural context of low- and middle-income countries, a breast cancer diagnosis may trigger shifts in family dynamics, where patients may distance themselves from their husbands due to stigma or the intention to protect their families, or even face the risk of their spouses remarrying. This dynamic instability of the marital relationship was not captured in the current study; thus, we cannot exclude the confounding effect of marital quality or stability on dyadic psychological distress.

Future research should expand the sampling scope to conduct multi-center surveys to improve representativeness. Additionally, future studies should incorporate the aforementioned relational variables at baseline and further explore marital transitions (e.g., separation, divorce) as special life events that may moderate the perception of financial toxicity and its dyadic interaction mechanisms.

Regarding methodology, although this study preliminarily explored the interaction between financial toxicity and psychological distress among patients and spouses, it did not deeply uncover the dynamic evolutionary pathways of these bidirectional relationships. Future studies could introduce parallel latent growth models or cross-lagged latent growth models to further analyze the longitudinal trajectories of mutual influence between couples, controlling for individual initial status and growth trends. This would allow for a more systematic and nuanced characterization of the coordinated change patterns and reciprocal mechanisms during the disease trajectory. Furthermore, integrating qualitative research could help elucidate the psychological and social interaction processes underlying the “actor effects” and “partner effects,” providing a more solid theoretical basis and practical guidance for developing couple-based psychosocial interventions.

## Conclusion

There is a mutual predictive relationship between financial toxicity and psychological distress in breast cancer patients and their spouses. The actor effect is significant. The financial toxicity of patients and their spouses can positively predict the psychological distress in the next stage, and the psychological distress of patients and their spouses can positively predict the financial toxicity in the next stage. The partner effect has partial significance, and the psychological distress of patients and their spouses can positively predict the psychological distress of each other in the next stage.

## Data Availability

The original contributions presented in the study are included in the article/supplementary material, further inquiries can be directed to the corresponding author.
